# The physician and professionalism today: challenges to and strategies for ethical professional medical practice

**DOI:** 10.1186/s12913-020-05884-1

**Published:** 2020-12-09

**Authors:** Jean-Pierre Unger, Ingrid Morales, Pierre De Paepe, Michel Roland

**Affiliations:** 1 Department of Public Health, Institute of Tropical Medicine, Nationalestraat 155, B-2000 Antwerp, Belgium; 2 Office de la Naissance et de l’Enfance, French Community of Belgium, Chaussée de Charleroi 95, B-1060 Brussels, Belgium; 3 Département de Médecine Générale, Université Libre de Bruxelles, Route de Lennik, 808, BP 612/1, B-1070 Brussels, Belgium

**Keywords:** Medical professionalism, Medical education, Medical research, Public health, Health management, Health policy

## Medicine in the twenty-first century

Imagine that physicians could multiply their impact on people’s health and improve community health whilst tailoring the delivery of care to each individual patient. The ambit of the present supplement is to say how, with a public health insight into medical practice and physicians’ professionalism.

But clouds obscure the future of the medical profession. Artificial intelligence (AI) threatens to make it obsolete. The commoditisation of care undermines ethics. Bureaucracy is infiltrating practice. Worldwide, cultures are turning materialistic. However, some physicians are resisting these trends. In spite of being well paid, the suicide rate of physicians in the United States is the highest of all occupations, almost twice the national average, and higher than in the military [1]. Taking decisions against one’s intimate convictions partly explains that [2].

To help doctors surmount this existential crisis and resist harsh intangible work conditions, this collection of articles gives them reasons to believe in the survival of medicine as a profession. To do so, the series elaborates on the changes needed in medical culture to keep medical practice as a sacred art, concentrating on what AI does not do or does not do well, especially ethical thinking. With such cultural changes, physicians would impose on AI design the function to support professional endeavour and undermine the opposite process that transforms professionals in technicians. To remain in control, the doctor’s intelligence, emotions, knowledge, communication, ethics, and creativity will have to surpass those of AI, not only in delivering biopsychosocial, ethical care but also in another, insufficiently explored domain that is, in improving collective health with clinical medicine.

Since taxes finance doctors, societies are entitled to demand them to optimise their impact on collective health whilst tailoring healthcare to the patient’s individual needs as much as possible. To meet the practicalities of such a paradoxical norm, the series advocates and delineates the practical and theoretical integration of clinical and public health practice to meet the challenges that medicine faces to survive as a profession, be they financial, political, managerial, or socio-cultural.

In practice, clinicians need to think and act whilst bearing in mind community health stakes - a duty, the importance of which has been clearly demonstrated by the Covid-19 crisis. Conversely, public health physicians must improve clinical healthcare whilst tackling population health risks. Updating the physician’s commitment and medical theory is necessary because dual clinical/public health medical practice is a scientific requirement to optimise the physician’s impacts on individual and collective health.

We adopted the following definitions in collective health.

1. According to WHO, “Public health refers to all organised measures (whether public or private) to prevent disease, promote health, and prolong life among the population as a whole.” (Available: http://www.who.int/trade/glossary/story076/en/) Public health relies on medical, epidemiological, socio-cultural, and environmental techniques. Historically, It has been formed from attempts to control epidemics. Public health addresses “organic” society (such as communities or trade-unions) incidentally, as a disease control resource.
2. By contrast, community medicine treats “organic” society as a goal per se, health being one of many issues to improve. Community medicine expands the realm of family medicine to social entities larger than the nuclear or extended family for more effective family medicine, just as family medicine broadens the realm of general practice clinical care for the same reason.
3. Preventive medicine aims to improve the health of high-risk groups defined by demographic and epidemiological characteristics (general population, infants, pregnant women, immune-compromised, the elderly, and others) whilst relying on health professionals and a medical, biopsychosocial rationale. Preventive medicine is thus by definition a branch of public health but one tightly connected to community and family medicine because of its reliance on physicians to deliver care.

If such joint practice sounds abstract, consider what doctors can do to potentiate clinical outputs. They can build and lead teamwork, reflect on practice, coach, educate, train, improve health service organisation, coordinate and evaluate health care, improve care accessibility, contribute to disease and health risk control, participate in public health programmes, delegate tasks, improve resource utilisation, do operational research, and lobby for more appropriate health policy design.

Physicians who (inter-)connect all or part of this to their clinical and public health practice could be called “manager physicians”. The term manager is traditionally reserved for persons entrusted with decision-making to achieve their institutions’ predetermined goals most efficiently, but our proposed definition assumes that professional ethics can prevail over institutional missions.

To use her/his knowledge and experience fully, any doctor can adopt the role of “manager physician”, an appealing role when education and research are professionally valued. In fact, any doctor has to adopt this role because the opportunity cost of not adopting it is to be measured in terms of avoidable suffering and mortality [3]. In sum, doctors ought to be “manager physicians” independently of or prior to the adoption of ad hoc institutional policies, thus possibly without being commissioned to be so, for the sake of professional ethics.

To explore the boundaries of medicine and public health, we analysed their (practical, epistemological, ethical, managerial, heuristic, investigative, and political) common facets separately.

The papers are titled as follows:

1. A Plea to Merge Clinical and Public Health Practices. Reasons and consequences
2. Integrating Medical and Public Health Knowledge – in Support of Joint Medical Practice.
3. In Defence of a Single Body of Clinical and Public Health Medical Ethics
4. Medical Heuristics and Action-Research. Professionalism versus science
5. Objectives, Methods, and Results in Critical Health Systems and Policy Research. Evaluating the healthcare market
6. Neo-Hippocratic Health Care Policies. Professional or industrial health care delivery? A choice for doctors, patients, and their organisations

## Patient and public involvement

Patients were not directly involved in the design and conduct of the present studies: the papers come out of a meta-analysis of the authors’ own experience and research.

In discussing the practice and theory of clinical medicine and public health, the series analyses the encounter of physicians and patients as follows: It uses the patients’ care quality demands as key yardsticks for professional behaviour, i.e., that care ought to be biopsychosocial, person-centred, and negotiated, and therefore highly individualised. This use of patient demand for setting health service standards is explicit throughout the analyses.

Besides, at some stage over the past 35 years, the following patients’, socio-political, and professionals’ organisations were unwittingly involved in the formulation of the concepts exposed here as they engaged in a dialogue with the authors and/or participated in their action-research: Health committees of first-line healthcare services in Congo (then called Zaire), Senegal, Burkina Faso, and Ecuador; Christian and Socialist mutual aid societies (Belgium); Plateforme Santé Solidarité (Belgium); Commisión Obrera de Bolivia; Sindicato Nacional de Trabajadores del Ministerio de Salud, Ecuador; Christian and Socialist Belgian trade unions; Fédération des maisons médicales (Belgium); Asociación Latinoamericana de Medicina Social; Royal Thai Association of Rural Doctors; Associação Brasileira de Saúde Coletiva; Entraide Fraternité Médicale Guinée; and Médecins du Monde, Belgium.

## The authors

This series of papers, intended primarily as guidance for physicians, draws on what the authors have learnt from their combined clinical and public health careers. In various ways, they have been involved in clinical, managerial, and/or public health practice; professional and scientific research; medical education; and health policy interventions for more than three to four decades.

In the course of their careers, Jean-Pierre Unger and Pierre De Paepe concentrated on the practice of physicians and inter-professional teams and researched healthcare policies. They used empirical learning and action-research to test strategies designed to improve medical professionalism with “non managed-care” techniques [4]; access to essential generic drugs (in Senegal) [5]; access to first-line health services (in Ecuador) [6]; the training and coaching of mid-level health managers (in Senegal) [7]; local health system organisation (in Senegal, Burkina Faso, and Niger) [8]; medical audits to enhance medical reflectivity and care coordination across institutional divides (in Belgium) [9]; family medicine-like healthcare practice by non doctors in LMICs [10]; the contribution of academic departments to the universal right to care (in Latin America and Belgium) [11]; public services strengthening with mother and child programmes (in Senegal) [12]; the national, publicly-oriented drug distribution system (in Burkina Faso) [13]; use of semi-quantitative epidemiological information to manage African local health systems [14]; symbolic motivation of health professionals in publicly-oriented health services; the effectiveness of malaria control (in Mali) [15]; GPs’ practice of family therapy in LMICs [16]; social assistance in medical practice (in Belgium) [17]; care coordination in Latin America [18]; and the control of physician and health professional burnout [2].

After practising clinical medicine in Bolivia and Brazil, Ingrid Morales shifted her focus, to epidemiology, nosocomial infection surveillance and control in academic and health service environments in Belgium and Europe in 1991. In 2011, she began a career in maternal and child health, guided by a concern to promote biopsychosocial care in preventive medicine and public health organisations. Ingrid is currently medical director of the maternal and child health programme of the French-speaking Community of Belgium (Office de la Naissance et de l’Enfance).

Michel Roland is a practising family doctor and professor emeritus who is concerned with the development of public health activities in medical practice. Whilst Ingrid, Jean-Pierre, and Pierre integrated clinical practice and culture in their public health practice, Michel did the opposite, integrating public health concerns in clinical practice and education, professional organisations, and government policies.

The authors came to question commercial clinical practice and pro-market health policies as their ethical, professional principles were at odds with the essence of care commoditisation. Being moral, their professional culture influenced their research objectives and methods, and so may well have generated a confirmation bias. They tried to circumscribe that risk by choosing sound research methodologies and taking a phenomenological perspective. To allow for critical appraisal, they ought to give the reader sufficient information not only on their methods but also on philosophical values. Over their professional life, the authors shared the following values

- They treated access to professionally delivered health care as a human right.
- For the sake of this right, they favoured solidarity and equity in care financing and health policies.
- They were convinced that giving the best possible health care depends not only upon the doctors’ professional skills and scientific knowledge, but also upon their emotions, in particular their self-effacement and ability to empathise with others.
- Finally, they promoted individual and community participation in health services for the sake of more effective care and the democratic functioning of public services.

The readers will judge the practical relevance of the research conclusions drawn, assess whether the authors’ social and professional commitment was sufficiently grounded in a concern for reflective methods and scientific accuracy, and if their science was sufficiently governed by ethics.

Although this series is devoted mainly to medicine, we hope that nurses, dentists, psychologists, and physiotherapists will judge the relevance of the medical concepts, analyses, and conclusions presented here to their professions.

## Data Availability

Data sharing is not applicable to this article as no datasets were generated or analysed during the current study.

## Abbreviations

AI: Artificial Intelligence
CME: Continuous Medical Education
GP: General Practitioner
LMIC: Low and Middle Income Countries
